# Advancing psychosocial disability and psychosocial rehabilitation research through large language models and computational text mining

**DOI:** 10.1017/gmh.2024.114

**Published:** 2024-12-13

**Authors:** Soheyla Amirian, Ashutosh Kekre, Boby John Loganathan, Vedraj Chavan, Punith Kandula, Nickolas Littlefield, Joseph R. Franco, Ahmad P. Tafti, Ikenna D. Ebuenyi

**Affiliations:** 1 School of Computing, University of Georgia, Athens, GA, 30602 USA; 2 Intelligent Systems Program, University of Pittsburgh, Pittsburgh, PA, 15213, USA; 3 Pace University, New York, NY 10038, USA; 4 School of Health and Rehabilitation Sciences, University of Pittsburgh, Pittsburgh, PA 15260, USA

**Keywords:** psychosocial disability research, psychosocial rehabilitation, computational text mining, large language models (LLMs)

## Abstract

Psychosocial rehabilitation and psychosocial disability research have been a longstanding topic in healthcare, demanding continuous exploration and analysis to enhance patient and clinical outcomes. As the prevalence of psychosocial disability research continues to attract scholarly attention, many scientific articles are being published in the literature. These publications offer profound insights into diagnostics, preventative measures, treatment strategies, and epidemiological factors. Computational text mining as a subfield of artificial intelligence (AI) can make a big difference in accurately analyzing the current extensive collection of scientific articles on time, assisting individual scientists in understanding psychosocial disabilities better, and improving how we care for people with these challenges. Leveraging the vast repository of scientific literature available on PubMed, this study employs advanced text mining strategies, including word embeddings and large language models (LLMs) to extract valuable insights, automatically catalyzing research in mental health. It aims to significantly enhance the scientific community’s knowledge by creating an extensive textual dataset and advanced computational text mining strategies to explore current trends in psychosocial rehabilitation and psychosocial disability research.

## Impact Statement

This study explores the potential of computational text mining algorithms along with large language models (LLMs) in advancing psychosocial disability and psychosocial rehabilitation research, discussing their capabilities in harnessing the large column of scientific articles in an automatic manner.

## 1. Introduction

The prevalence of mental disorders is on the rise worldwide, leading to a growing concern regarding psychosocial disability. According to the 2019 Global Burden of Disease study, mental disorders are among the top 10 leading causes of global disease burden, with disability-adjusted life years (DALYs) attributed to mental disorders increasing from 3.1% to 4.9% between 1990 and 2019 (Collaborators, GBD 2019 Mental Disorders, et al., 2022). A cross-sectional study from India reported a prevalence of psychosocial disability at 4.8%, with 75% of participants with psychological distress experiencing comorbid functional impairments (Mathias et al., 2018). Despite the already recognized impact of mental health disorders on disability, societal and health system acceptance remains challenging, with individuals with psychosocial disabilities facing unique challenges due to historical and societal perceptions (Ringland et al., 2019). Psychosocial disability encompasses various mental health conditions, resulting from a combination of factors including stigma, discrimination, and exclusion (WHO, 2015a). While serious mental illnesses such as schizophrenia, schizoaffective disorder, and bipolar disorders are traditionally associated with disability, common mental health conditions like depression or anxiety can also lead to impairment in social and occupational functioning (Ebuenyi et al., 2019).

Users and survivors of psychiatry in Kenya prefer the term psychosocial disability, which acknowledges the impact of socioenvironmental factors on mental health, highlighting the rights of affected individuals to define their experiences and the importance of addressing social determinants of health (USPKenya, 2017). While psychosocial disabilities may also be described as mental or psychiatric disabilities, the use of psychosocial disabilities highlights the impact of socioenvironmental factors on the experience and impact of mental health conditions. Psychosocial rehabilitation aims to facilitate individuals disabled by mental disorders to achieve optimal independent functioning in the community, offering tailored supports such as access to mental health services, housing, employment, and education (WHO, 2015b, 1996; Yildiz, 2021). The World Health Organization (WHO) endorses psychosocial rehabilitation to improve the quality of life of people with mental health conditions (WHO, 2015b). Yet, globally, access and quality of psychosocial rehabilitation services continue to be dismal, especially in low-income/resource settings. Saha et al. (2020) argue that despite the high prevalence of mental health conditions, a limited number of psychosocial rehabilitation centers exist. These have health and socioeconomic implications for affected individuals. The dissonance in language and understanding of what psychosocial disability entails and the scope of rehabilitation needed also contribute to challenges faced by affected individuals. Understanding the scope of the problem and using evidence-based methods and technologies such as artificial intelligence may provide further insights into the magnitude of the problem and options for addressing the challenges. This study aims to use artificial intelligence (AI) to current scientific knowledge on psychosocial disability and rehabilitation to create available datasets with which to drive scientific research and policy interventions in this area of study.

**Figure 1.**
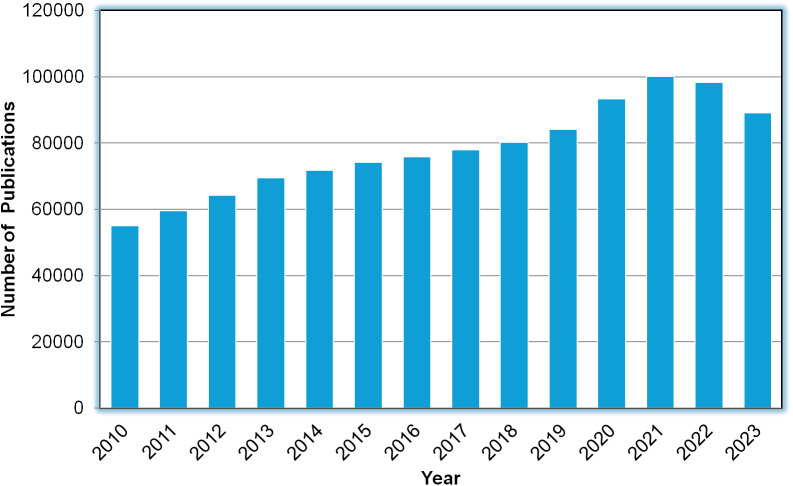
The number of publications in psychosocial rehabilitation and mental disability research available at PubMed over the last 14 years. The results obtained by submitting a query on PubMed: (“Mental Disorders”[Mesh] OR “Mentally Ill Persons”[Mesh] OR “Persons with Mental Disabilities”[Mesh] OR “severe mental”[tiab] OR psychosis[tiab] OR psychoses[tiab] OR psychotic[tiab] OR schizo*[tiab] OR bipolar*[tiab] OR “mental disab*”[tiab] OR “mentally disab*”[tiab] OR “psychiatric disab*”[tiab] OR “psychosocial disab*”[tiab] OR “psycho-social disab*”[tiab] OR “major depress*”[tiab] OR “anxiet*”[tiab] OR “depressive”[tiab] OR Rehabilitation, Psychiatric[MeSH Terms] OR Mental Health Rehabilitation[MeSH Terms] OR Health Rehabilitation, Mental[MeSH Terms] OR Rehabilitation, Mental Health[MeSH Terms] OR Psychosocial Rehabilitation[MeSH Terms] OR Rehabilitation, Psychosocial[MeSH Terms] OR Psychosocial Care[MeSH Terms] OR Care, Psychosocial[MeSH Terms] OR Cares, Psychosocial[MeSH Terms] OR Psychosocial Cares[MeSH Terms]) AND 2010/01/01:2023/12/31[Date – Publication].

Utilizing evidence-based methods such as AI combined with a large column of scientific literature holds promise in enhancing our understanding of psychosocial disability and rehabilitation, thus informing future research and policy interventions in this critical area (Ebuenyi et al., 2019). In recent years, there has been a significant amount of biomedical literature with a notable increase in publications related to mental disorders and disabilities studies, including psychosocial rehabilitation, drug developments, and therapies. Figure 1 illustrates the exponential growth in the number of journal and conference papers in these areas from 2010 to the present. The total volume of publications during this period reached approximately 1,093,206 articles.

Scientific research articles indexed by PubMed (*Pubmed*) are often produced using standardized and rigorous methodologies, making them invaluable sources for knowledge discovery (Amirian et al., 2023b). This data repository includes a considerable number of publications focused on the study of mental health, attracting numerous biomedical researchers who engage in various research endeavors to aim at discovering, analyzing, and monitoring mental disorders and psychosocial disability and rehabilitation (Gatchel et al., 2006). However, navigating through this large volume of literature poses a significant challenge to innovative approaches to extracting meaningful insights efficiently, and this is where AI offers the potential to cope with the task.

AI-powered text mining techniques equipped with large language models (LLMs) applied to scientific literature, particularly utilizing resources like PubMed, have emerged as promising tools for extracting relevant information and uncovering hidden patterns within this large-scale corpus of scientific literature. By harnessing computational algorithms and natural language processing (NLP) methods, researchers can sift through extensive amounts of textual data, identify key concepts and establish connections between different studies and findings related to mental health and mental disability. This approach synthesizes existing knowledge and provides a foundation for generating new hypotheses and directing future research paths in this field.

While psychosocial rehabilitation and psychosocial disability research have been a longstanding topic in healthcare, the use of computational text mining and LLMs on scientific articles’ knowledge discovery in mental health has been minimal so far. Thus, the motivation of this work is to study advanced computational text mining, such as word embeddings and in particular, LLMs, to fulfill the following objectives: (1) to extract current knowledge and high-quality information about psychosocial rehabilitation and psychosocial disability research using large-scale scientific abstracts published in PubMed, (2) to utilize and adapt LLMs in a large-scale fashion by the use of cloud services, and (3) to provide better insights and tendencies in large-scale biomedical text analytics in mental health settings.

## 2. Related work

The intersection of computational linguistics with mental health research over recent years has brought in a new era in the diagnostics, treatment, and comprehension of mental health conditions and disabilities. This novel synergy aims at enhancing the granularity of diagnostic and therapeutic avenues while bridging the chasm between the vast reservoir of public inquiries and the structured repository of mental health knowledge. The ongoing research in this field can be meticulously categorized into three pivotal streams: Text Mining on Mental Health/Disability, NLP on Mental Health/Disability, and LLMs on Mental Health/Disability, each delineating a unique facet of the computational linguistic approach to mental health.

### 2.1. Text mining on mental health/disability

Text mining, as a foundational pillar of this trial, serves as an instrumental channel in disseminating mental health knowledge. The work by Park et al. (2021) exemplifies the instrumental role of text mining in deciphering the nuances of public queries on mental health across online platforms. This initiative sheds light on both the commonalities and divergences in public inquiries about mental disorders, while also paving the way for customizing public health communications and interventions. Furthermore, the application of text mining in extracting mental health disorders from the narratives of domestic violence by another study (Karystianis et al., 2018) unfolds a novel view of understanding the psychological ramifications of domestic abuse. This venture into the psycholinguistic dimensions of trauma narratives significantly contributes to the forensic and therapeutic domains by offering insights into the intersection of language, trauma, and psychological well-being. Additionally, the utility of text mining in parsing through electronic health records to validate diagnoses of major depressive disorders (Wu et al., 2020) presents the critical role of text analytics in bolstering the diagnostic framework for mental health conditions. This approach enhances the precision and reliability of diagnoses while highlighting the potential of text mining in streamlining health records analysis, thereby facilitating a more nuanced and comprehensive understanding of mental health conditions.

### 2.2. NLP on mental health/disability

Building upon the foundational insights offered by text mining, NLP further extends the analytical capabilities into the domain of mental health and disability determination. The innovative framework introduced for disability determination using NLP (Zirikly et al., 2022) showcases the remarkable potential of computational linguistics in deconstructing complex medical narratives into actionable insights. This not only augments the efficiency of the disability determination process but also introduces a layer of precision and nuance that was previously unattainable. Similarly, the convergence of machine learning and NLP in mental health research, as explored in a systematic review (Le Glaz et al., 2021), unveils the diverse applications of these technologies in understanding, diagnosing, and treating mental health conditions. The predictive modeling capabilities of machine learning classifiers, as discussed in another study (Dristy et al., 2022), offer a tantalizing glimpse into the future of diagnostic tools that could leverage linguistic patterns to predict mental health statuses. This innovative merger of NLP and machine learning heralds a new dawn in mental health research, promising tools that are more accurate and efficient, capable of preempting the onset of mental health issues through predictive analytics.

### 2.3. LLMs on mental health/disability

The advent of LLMs in the field of mental health research marks the latest evolution in the application of computational linguistics. The study by Zhang et al. (2023) that discusses AI’s role in rehabilitation medicine through LLMs epitomizes the cutting-edge potential of these models in transforming the therapeutic landscape. This highlights the efficacy of LLMs in clinical settings and also opens up new avenues for personalized and accessible mental health interventions. Furthermore, the scalability of psychological services through AI-based models as explored in subsequent studies (Lai et al., 2023; Jin et al., 2023) signifies a monumental shift toward democratizing mental health services. By leveraging the computational prowess of LLMs, these studies endeavor to transcend geographical and economic barriers, making mental health support more accessible and inclusive. Innovative approaches like MentaLLaMA and Chat Counselor (Yang et al., 2023; Liu et al., 2023) further illustrate the potential of conversational models and social media analytics in providing real time, interpretative support for individuals grappling with mental health issues. This announces a new era of digital mental health interventions, emphasizing the role of LLMs in crafting a more empathetic and responsive mental health ecosystem.

This section collectively presented the transformative potential of textual content mining, NLP, and LLMs in advancing intellectual health research and practice. With this recognition in mind, our research focuses on employing computational text mining methods, specifically employing advanced techniques like word embedding and LLMs, to extract meaningful insights from the extensive body of scientific literature found on PubMed (*Pubmed*), concerning psychosocial rehabilitation and mental disability. Our goal is to establish a valuable foundation for future research in leveraging computational methods to enhance understanding and interventions in psychosocial disability research.

## 3. Materials and methods

**Figure 2.**
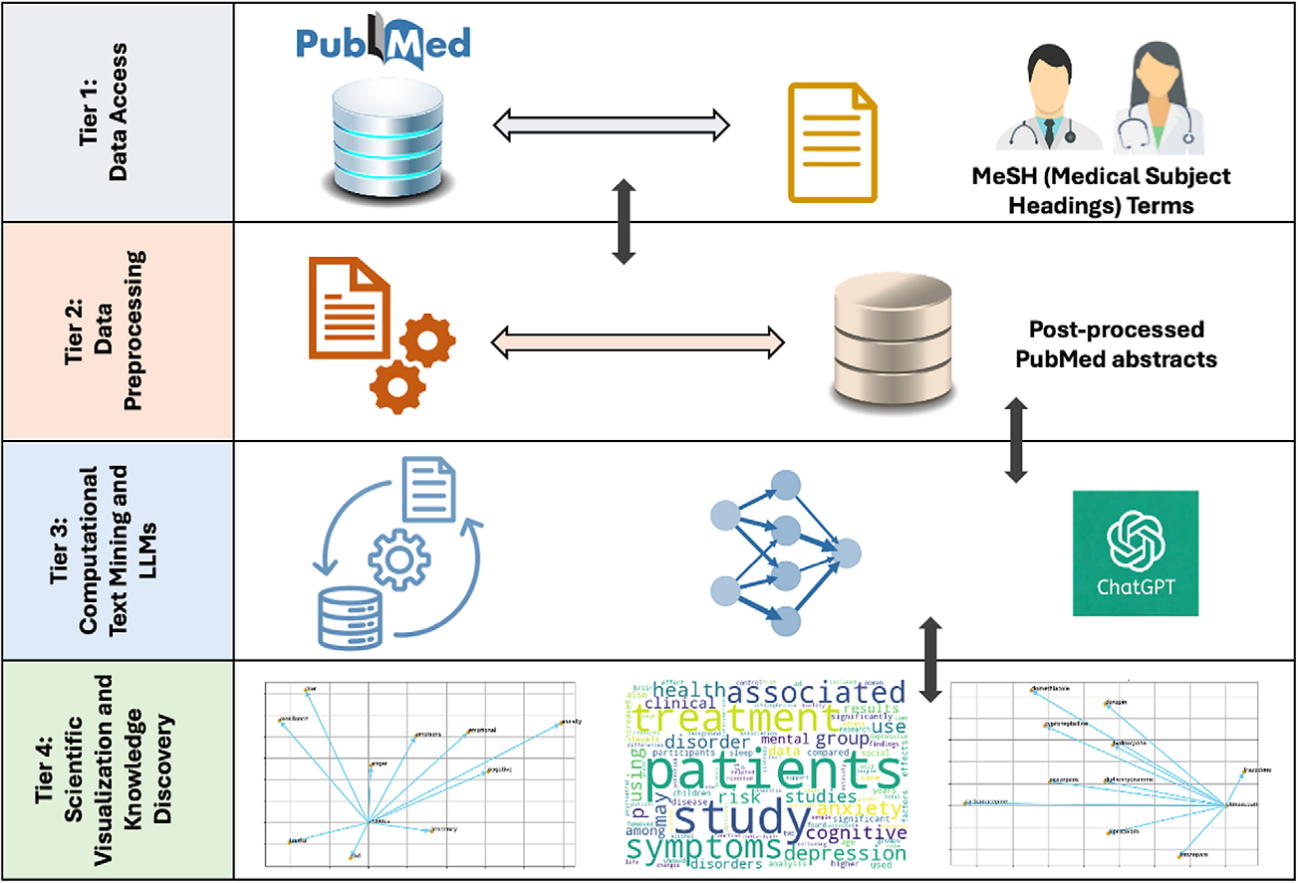
The underlying tiers of the proposed computational text mining framework.

The proposed computational text-mining framework for psychosocial rehabilitation and mental disability research is depicted in Figure 2. This section provides a detailed explanation of the underlying tiers within this framework.

### 3.1. Tier 1: Dataset access

We shall begin with the dataset that was computationally assembled through PubMed (*Pubmed*). To assemble a textual dataset from PubMed abstracts, we used Medical Subject Heading (MeSH) terms restricting our query to search within the title or abstract of the articles. We defined the publication date within the range from January 1, 2000, to December 31, 2023. Our search spans two categories, including “psychosocial/mental disability” and “psychosocial rehabilitation.” Regarding “psychosocial/mental disability,” we used the following PubMed query to collect relevant abstracts from the scientific articles:

**PubMed query for psychosocial or mental disability**

(“Mental Disorders”[Mesh] OR

“Mentally Ill Persons”[Mesh] OR

“Persons with Mental Disabilities”[Mesh] OR

“severe mental”[tiab] OR

“psychosis”[tiab] OR

psychoses"[tiab] OR

“psychotic”[tiab] OR

“schizo*”[tiab] OR

“bipolar*”[tiab] OR

“mental disab*”[tiab] OR

“mentally disab*”[tiab] OR

“psychiatric disab*”[tiab] OR

“psychosocial disab*”[tiab] OR

“psychosocial disab*”[tiab] OR

“major depress*”[tiab] OR

“anxiet*”[tiab] OR

“depressive”[tiab]) AND

2000/01/01:2023/12/31[Date - Publication]

To collect abstracts associated with “psychosocial rehabilitation,” we used the following PubMed query:

**PubMed query for psychosocial rehabilitation**

Rehabilitation, Psychiatric[MeSH Terms] OR

Mental Health Rehabilitation[MeSH Terms] OR

Health Rehabilitation, Mental[MeSH Terms]

Rehabilitation, Mental Health[MeSH Terms] OR

Psychosocial Rehabilitation[MeSH Terms] OR

Rehabilitation, Psychosocial[MeSH Terms] OR

Psychosocial Care[MeSH Terms] OR

Care, Psychosocial[MeSH Terms] OR

Cares, Psychosocial[MeSH Terms] OR

Psychosocial Cares[MeSH Terms] AND

(“2000/01/01”[Date - Publication]: “2023/12/31”

[Date-Publication])

The MeSH terms used for psychosocial disability and rehabilitation were adapted from the ones developed by a medical information specialist and previously used in two different published reviews (Ebuenyi et al., 2018; Ebuenyi et al., 2023).

### 3.2. Tier 2: Dataset preprocessing

We implemented a normalization method to create consistency within the textual material. This meant changing every word to lowercase to remove any inconsistencies that might have resulted from different capitalization. We also eliminated all unnecessary punctuation, unusual characters, and numbers that did not contribute to the data. We wanted to make it easier to analyze and interpret the dataset by standardizing the wording in this way.

After normalization, the text was divided into discrete words, or tokens, by a process known as tokenization. We concurrently implemented stop-word removal to ensure that the dataset consisted mostly of content words important to the medical context. By eliminating common stop words—such as “the,” “and,” and “is”—which lack specific meaning in the context of medical literature, we aimed to enhance the relevance and specificity of the dataset for our analysis.

Precise regular expression (regex) patterns were designed to locate and remove unnecessary textual segments. This included eliminating author affiliations, bibliographic information, and other metadata that can distort or confuse the analysis. We attempted to streamline the dataset and ensure that it contained just the most relevant textual content for our study goals by using advanced regex patterns that were specifically designed to collect and remove unnecessary information.

By systematically carrying out these operations, we attempted to clean and organize the dataset, creating a solid basis for further analysis. We attempted to improve the quality and usefulness of the dataset for obtaining practical clinical insights by standardizing the textual data, focusing on pertinent terms in its content, and removing unnecessary information.

### 3.3. Tier 3: Computational text mining and LLMs

This section will delve deeper into our proposed strategy, utilizing computational text mining methods, in particular word embeddings and LLMs. Word embeddings capture semantic relationships between words, enabling meaningful analysis beyond frequency-based approaches, such as TF-IDF (term frequency-inverse document frequency). LLMs, such as those based on transformer architectures, can generate coherent text and have been validated extensively in NLP tasks. They offer robust capabilities for summarization, categorization, and trend analysis in large-scale biomedical text datasets (Chen et al., 2023; Amirian et al., 2023a).

#### 3.3.1. Word embeddings: Word2vec and GloVe

Word2vec (Mikolov et al., 2013) and GloVe (Pennington et al., 2014) are neural network-based algorithms for generating word embeddings, which represent words as continuous vectors in a multidimensional space based on their contextual usage within a corpus. These embeddings have demonstrated significant utility across various tasks, including named entity recognition (NER) (Nozza et al., 2021; Naseem et al., 2020), text classification (Sun et al., 2024; Singh et al., 2022), and sentiment analysis (Zhu and Samsudin, 2024; Suhartono et al., 2023).

Rather than only encoding word frequencies, these models also encode information about word order, syntax, and semantics within the corpus. The primary objectives of word embeddings, such as Word2vec and GloVe, include: (1) serving as input features for machine learning algorithms, (2) facilitating nearest neighbor search operations in the embedding space, and (3) aiding in the visualization of semantic relationships between different words in the context.

While GloVe operates on co-occurrence statistics to generate word embeddings, Word2vec employs a context-based approach and is commonly used as a predictive model. Word2vec encompasses two distinct learning strategies: Continuous Bagof-Words (CBOW) and Skip-gram. CBOW predicts a target word given its context, whereas Skip-gram predicts the context given a target word. Both models are trained to minimize specific loss functions (such as hierarchical softmax, full softmax, or noise contrastive estimation) during the training process. For example, using the word2vec skip-gram model, one loss function can be the full softmax, and then, the very final output layer will apply softmax to estimate the probability of predicting the output word 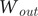 given 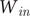 , as follows:(1)

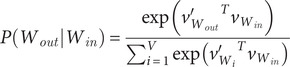

where the embedding vector of every single word is defined by the matrix *W* and the context vector is determined by the output matrix 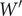 . Given an input word as *W_in_*, it identifies the corresponding row of matrix *W* as vector 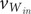 , the embedding vector, and its corresponding column of 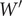 as 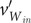 , the context vector. In contrast, when the total size of the vocabulary is immense, a loss function such as hierarchical softmax would be a better option.

In this work, we utilized the skip-gram model since it suits large-scale data. GloVe however works differently. Instead of extracting the embeddings from a neural net, the embeddings are optimized directly in a way that the dot product of two word vectors would be equal to the log of the frequency the two words will occur near each other. GloVe defines the cooccurrence probability as follows:(2)

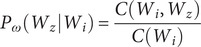



Here, 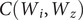 counts the co-occurrence between two words 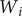 and 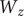 . We employed 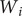 and 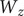 , to differ than 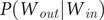 , which is presented in equation 1. For example, if two terms as “chlorpromazine” and “amisulpride” occur close to each other 1500 times in a given corpus, then *Vec* (*chlorpromazine*) ‧ *Vec* (*amisulpride*) = *log* (1500). This drives the vectors to encode the frequency distribution of which words lie near others.

The scope of the present work does not allow for an in depth exploration of word embedding strategies; interested readers are thus referred to (Johnson et al., 2023; Biswas and De, 2022; Sivakumar et al., 2020) for further reading.

#### 3.3.2. Large language models

Here, we aimed to use the latest version of ChatGPT, ChatGPT-4o, to answer questions based on the information contained in a sample of 20 PubMed abstracts and evaluate its performance. To avoid fine-tuning ChatGPT-4o, we utilized retrieval augmented generation (RAG).

##### 3.3.2.1. Preprocessing abstracts

Given the large number of abstracts available, we randomly selected 20 PubMed abstracts to construct a small database of information to see how well ChatGPT-4o can do at answering questions about the provided abstracts. To do this, each abstract was converted to an embedding and stored in a vector database. Each of the embeddings were constructed using OpenAI’s Embedding API. To be compatible with ChatGPT-4o, we utilized text embedding- ada-002.

##### 3.3.2.2. Communicating with ChatGPT

To communicate with ChatGPT-4o, we developed a small user interface that allows the user to prompt ChatGPT with a question related to the small database of abstracts. When ChatGPT was first prompted, we retrieved all documents related to the users’ prompt by using maximal marginal relevance. We only retrieved up to *k* most relevant documents and then returned the top 2 from that. These top 2 documents were then provided to ChatGPT as context for the question.

Once the relevant information to the question was obtained, we constructed the prompt to send to ChatGPT. This prompt consists of three key components: a system message, the user prompt, and the provided context. The system prompt was designed to specifically tell ChatGPT what its domain specific role is and the instructions to follow. Specifically, we provided ChatGPT-4o with the following system message: *You are a research scientist studying psychosocial rehabilitation and mental disability. Use only the provided context to answer the question. If you are unable to answer the question using only the provided context, say ‘I do not know.’.* This system message specifically instructs ChatGPT to use only the provided information and say ‘I do not know’ if it cannot construct one from the provided context.

After providing the prompt and context, we sent the system role, prompt, and context to ChatGPT using OpenAI’s API to synthesize a response to the question provided by the user. As provided in the system role, if ChatGPT cannot synthesize an answer from the provided context, it will return a message stating that it does not know the answer. To avoid hallucination, we use a minimum temperature value of 0.1.

##### 3.3.2.3. Explainability

**Figure 3.**
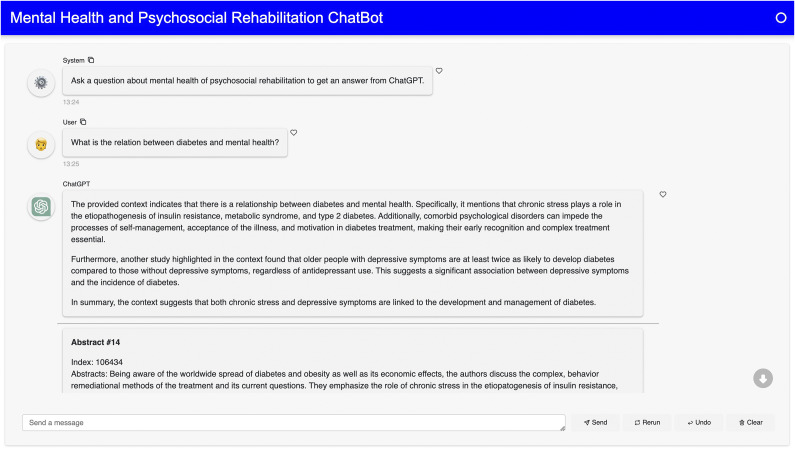
A response from ChatGPT for the question “What is the relation between mental health and diabetes?”

**Figure 4.**
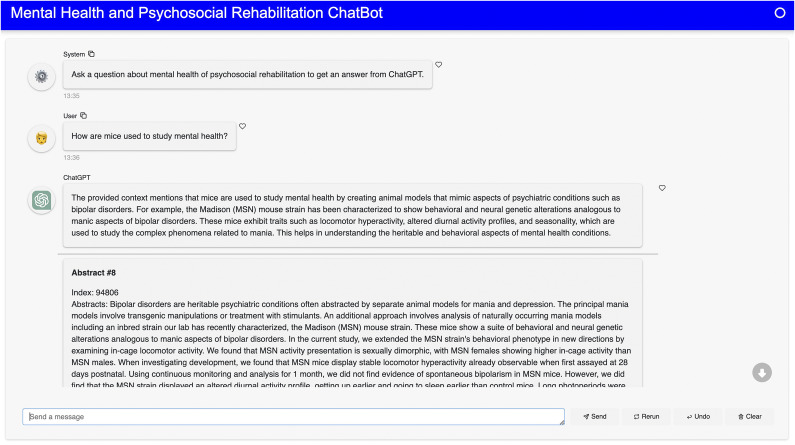
A response from ChatGPT for the question “How are mice used to study mental health?”

**Figure 5.**
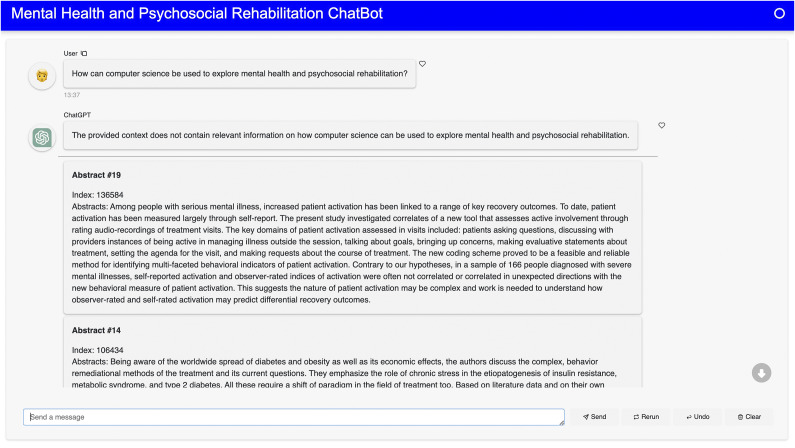
A response from ChatGPT for the question “How can computer science be used to explore mental health and psychosocial rehabilitation?”

To ensure that ChatGPT was synthesizing relevant and factual information to a user’s prompt, we included all relevant abstracts used to create the answer given. This allowed the user to see what abstracts were relevant to their question, what was being given to ChatGPT, and whether the answer was true. Figures 3, 4, and 5 provide three examples of prompts sent to ChatGPT, two where it was successful in generating a response, and one where it could not.

##### 3.3.2.4. Testing reliability and trustworthiness

To test how well ChatGPT-4o did with answering questions we asked it, we constructed a set of synthetic test questions and ground truths using the Ragas library.^1^ These questions consisted of three categories: simple, reasoning, and multi-context. Simple questions were systematically generated from the provided text documents, reason questions were rewritten in a way that enhances the need for the LLM to reason, in some way, when answering the question, while multi-context rephrases questions to make it necessary to use pieces of related information to formulate an answer. For our setup, we generated 20 synthetic questions, where 50% were simple questions, 25% were reasoning questions, and 25% were multi-context questions. To evaluate the responses, we utilized context precision and recall, faithfulness, answer relevance, and aspect critiques, including hallucination, maliciousness, correctness, coherence, and conciseness.

### 3.4. Tier 4: Scientific visualization and knowledge discovery

This tier mainly focuses on scientific visualization and knowledge discovery, where it aims to transform the insights gained from computational text mining and LLM analysis into easily interpretable and visually engaging representations. Through this tier, we leverage various visualization components to present the extracted knowledge and discovered patterns from the PubMed abstracts related to mental health.

One aspect of our visualization strategy involves constructing thematic maps illustrating the interconnectedness and clustering of key concepts within the retrieved abstracts. With that, we uncover the underlying structure of mental health research literature, identifying prevalent themes, and mapping the relationships between them. Overall, Tier 4 serves as a vital component in our effort to translate computational findings into actionable knowledge, facilitating a deeper understanding of psychosocial rehabilitation and psychosocial disability research through informative visual representations.

## 4. Results

### 4.1. Implementation and experimental setup

The implementation employed Python programming language and its libraries. The data preprocessing steps, encompassing normalization, tokenization, stop-word removal, and regex-based pattern matching for eliminating extraneous textual segments, were executed through Python scripts in a Google Colab environment. The integration of the Gensim library in Python facilitated the implementation of word embedding techniques, such as Word2Vec and GloVe.

Initially, the standard Google Colab platform was utilized as the testbed, offering a convenient cloud-based platform for prototyping and executing the Python scripts without the need for local setup and configuration. However, due to the substantial dataset size of approximately 3 GB, the system encountered limitations in terms of available RAM resources. To overcome this constraint, the project was transitioned to the Google Colab Pro version, which provided access to enhanced computational resources, like GPU v100 that accelerated the storage capacity and processing power.

### 4.2. Scientific visualization

Scientific visualization aims to represent vast and multi-dimensional datasets using charts, graphs, and images. The overarching goal of scientific visualization here is to enhance comprehension and insight into the PubMed data under investigation.

#### 4.2.1. Word similarities

Word similarities, a fundamental concept in NLP, play a pivotal role in understanding the semantic relationships between words within textual data. Word2vec (Mikolov et al., 2013) and GloVe (Pennington et al., 2014) have revolutionized this field by enabling the quantification of these relationships in high-dimensional vector space models. By visualizing these relationships, researchers can identify patterns and uncover latent semantic structures within scientific literature or experimental data.

**Figure 6.**
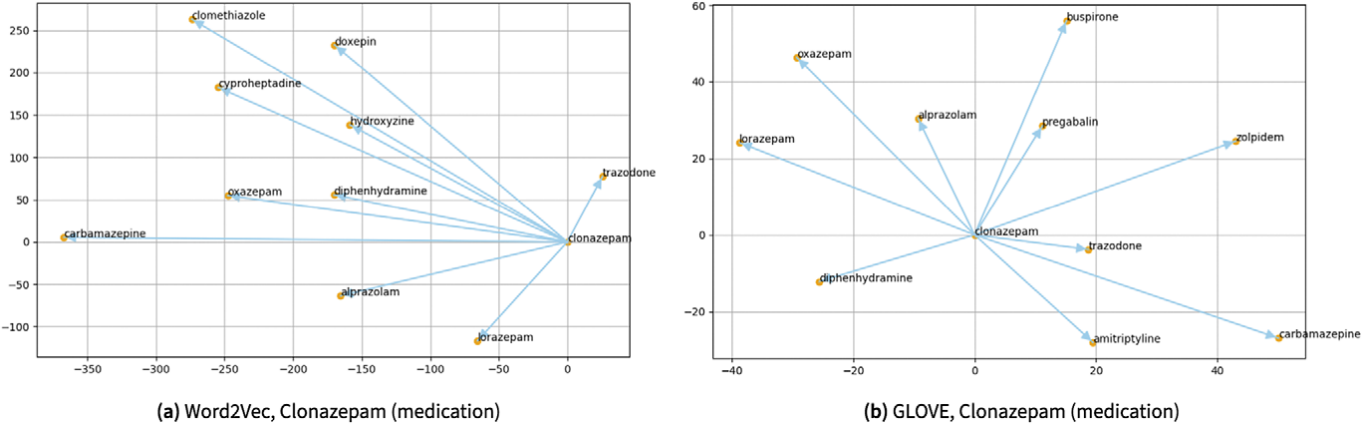
The scientific visualization results obtained by searching word similarities using the trained Word2Vec and Glove algorithms for “clonazepam” a benzodiazepine as one of the possible medications for anxiety and/or seizure disorders. One can see almost all terms presented here are associated with anxiety, depression, or panic disorders, such as “lorazepam,” “oxazepam,” “trazodone,” and “alprazolam.”

This section demonstrates a list of scientific visualizations focusing on word similarities within three contexts including medication, clinical symptoms, and rehabilitation strategies. With that, Figure 6 presents the scientific visualization results obtained by searching word similarities using the trained Word2Vec and Glove algorithms for “clonazepam,” as one of the possible medications linked to mental health issues is illustrated in Figure 6.

**Figure 7.**
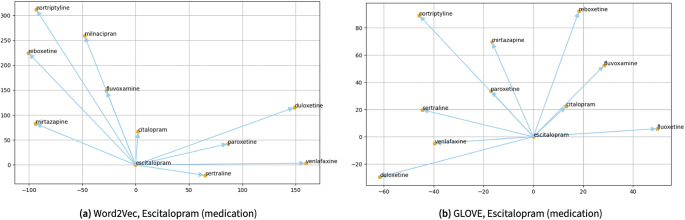
The scientific visualization by searching word similarities using the trained Word2Vec and Glove algorithms for “escitalopram” an anti-depressant, as one of the mental health-related medications for depression. One can see almost all terms presented here are associated with mental health medications, such as “citalopram,” “sertraline,” “reboxetine,” and “mirtazapine.”

**Figure 8.**
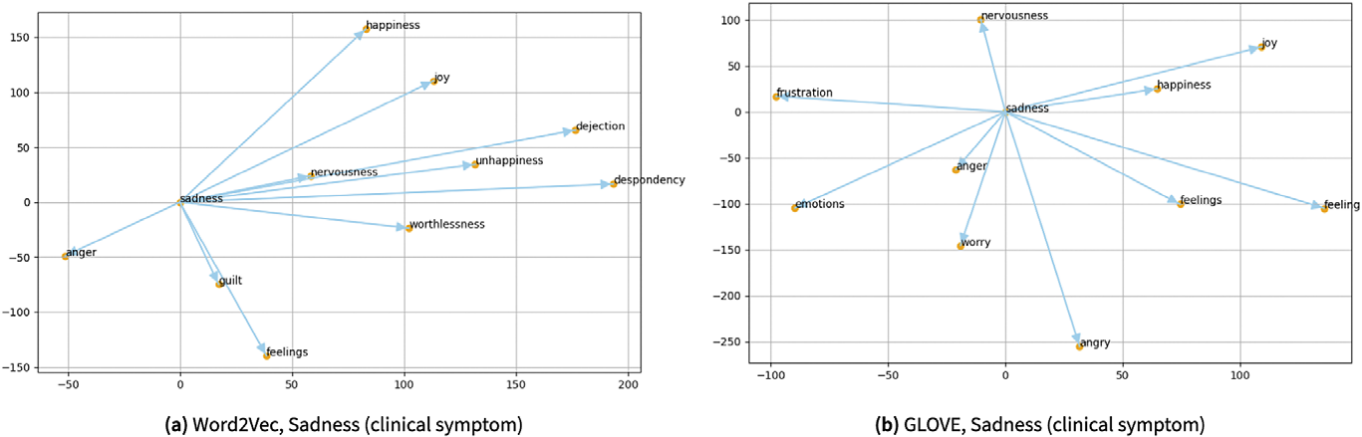
The scientific visualization by searching word similarities using the trained Word2Vec and Glove algorithms for “sadness,” as one of the mental health-related clinical symptoms. One can see almost all terms presented here are associated with subjective symptoms common in mood disorders, such as “anger,” “worry,” “unhappiness,” and “despondency.”

Similarly, the scientific visualization results by searching word similarities using the trained Word2Vec and Glove algorithms for a medication, namely “escitalopram,” is illustrated in Figure 7. Furthermore, Figure 8 shows scientific visualization results by searching word similarities using the trained Word2Vec and Glove algorithms for a clinical symptom of “sadness,” where it also demonstrates a correlation among “sadness” with other clinical symptoms, such as “anger,” “worry,” “guilt,” and “nervousness.”

**Figure 9.**
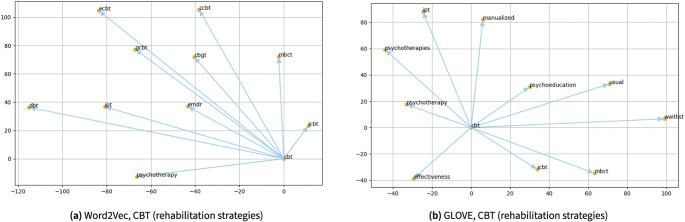
The scientific visualization by searching word similarities using the trained Word2Vec and Glove algorithms for “CBT,” a form of psychotherapy for different mental health conditions such as depression and anxiety orders. One can see almost all terms presented here are different forms of psychotherapy and variants of CBT such as “gCBT,” “cCBT,” and “iCBT.” This somehow illustrates a limitation within the current work.

Moreover, Figure 9 demonstrates scientific visualization results by searching word similarities using the trained Word2Vec and Glove algorithms for a rehabilitation strategy, namely “cognitive behavioral therapy (CBT).”

#### 4.2.2. Word clouds

Word Clouds are essential tool sets for visualizing word frequencies and relationships in a corpus. They condense complex textual data into easily understandable visuals, with word size reflecting frequency or importance. In scientific visualization, Word Clouds offer a quick, intuitive way to identify key terms, patterns, and clusters within large textual datasets, aiming to extract insights and explore data complexity.

**Figure 10.**
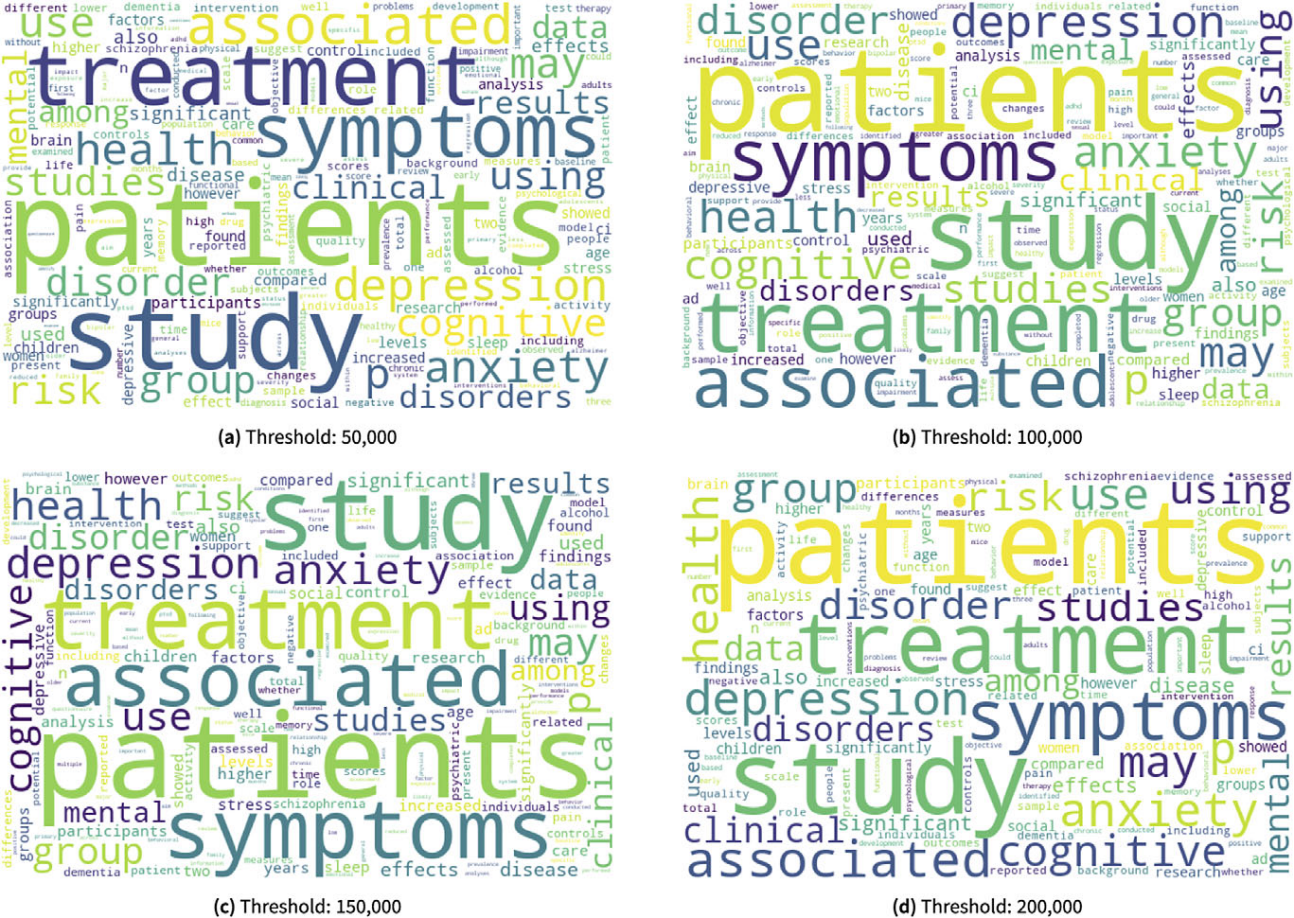
The word clouds using the entire abstracts collected through this study. Different thresholds were used to produce different word clouds from 50,000 to 200,000 in increments of 50,000, as shown in (a), (b), (c), and (d) respectively. Only words that occurred greater than or equal to the threshold were considered for each word cloud. The top 100 words are then used to generate the word cloud.

The word cloud representations obtained using the entire abstracts collected in this study is illustrated in Figure 10. One can see the most frequent words using different thresholds are “patients,” “treatment,” “symptoms,” and “study.” The prevalence of these words may indicate a strong emphasis on understanding patient experiences, interventions, and the manifestation of symptoms within the large body of the current PubMed abstracts. “Patients” in Figure 10 highlights the central focus perhaps on individuals receiving care or participating in research, suggesting a patient-centered approach, while the word “treatment” underscores a significant interest in therapeutic interventions or treatment plans and strategies. Furthermore, the word “symptoms” could indicate a comprehensive investigation into the clinical manifestations or indicators of psychosocial disability within the entire study. Finally, the repetition of “study” highlights a self-referential focus, potentially indicating an emphasis on methodological considerations and/or the exploration of the study’s design and outcomes.

The next level of the top frequent words in Figure 10 are “depression,” “anxiety,” “disorder,” and “cognitive.” One can observe that the inclusion of “depression,” “anxiety,” “disorder,” and “cognitive” as prominent terms in the word cloud signals a deeper exploration into specific psychosocial phenomena within the study. The prevalence of “depression” and “anxiety” highlights a significant focus on emotional well-being, suggesting a comprehensive examination of mental health challenges experienced perhaps by individuals with psychosocial disabilities. Moreover, the inclusion of “disorder” indicates an investigation into various psychiatric conditions, maybe reflecting efforts to better categorize or understand the diverse range of mental health presentations within the study population. Finally, the high frequency of the word “cognitive” could demonstrate an additional dimension of inquiry, potentially highlighting an exploration of cognitive functioning, deficits, or interventions aimed at addressing cognitive impairments in individuals with psychosocial disabilities.

### 4.3. Experimental validation

We validated the responses generated by ChatGPT-4o with the assistance of our experts and subsequently assessed their reliability, trustworthiness, and explainability for context retrieval.

#### 4.3.1. Domain-experts-in-the-loop

To ensure a comprehensive evaluation of ChatGPT-4o, we involved four domain experts in the validation process. This approach aimed to assess the system’s responses from multiple perspectives, including its alignment with human-like soft skills and expertise. We designed a detailed questionnaire to evaluate ChatGPT-4o’s performance on mental-health-related questions derived from 10 mental-health-related abstracts collected from our proposed dataset through PubMed, with 20 questions. The questions were categorized into three types: (1) simple questions, (2) reasoning questions, and (3) multicontext questions.

All four domain experts were tasked with reviewing the AI generated responses and marking their agreement on a scale of *Agree*, *Disagree*, and *Not Applicable.* To quantify the alignment between the domain experts’ assessments and ChatGPT-4o’s responses, we employed the Kappa measure (Eugenio and Glass, 2004). The Kappa score measures the level of agreement between raters beyond what would be expected by chance, thus providing insight into the reliability of the AI system’s performance compared to expert judgment. The Kappa scores calculated for this evaluation demonstrated an average observed agreement of 0.80. This result indicates substantial agreement between the domain experts and ChatGPT-4o, reflecting that the AI system’s responses were closely aligned with domain expert evaluations.

By incorporating this method, we addressed the need for a deeper evaluation of AI systems, beyond traditional accuracy metrics, and demonstrated how the AI’s performance aligns with human expertise and judgment meaningfully. Furthermore, involving domain experts allowed us to capture qualitative insights that are not solely evident from quantitative metrics. Experts provided valuable feedback on the contextual appropriateness and depth of the AI responses, highlighting areas where the AI performed well and where it could improve. This qualitative assessment helps to bridge the gap between AI performance and human-like interaction, underscoring the AI’s ability to engage in complex, context-sensitive dialogs and providing a more holistic view of its capabilities.

#### 4.3.2. Reliability, trustworthiness, and explainability

We evaluated the reliability, trustworthiness, and explainability of the RAG pipeline using simple and synthetic questions, which were created through a two-step process: (1) retrieval evaluation, and (2) response evaluation. These results are provided in Table 1. For context retrieval, we evaluated context precision and context recall. Context precision measures whether the relevant contexts provided by the retrieval pipeline rank higher than other contexts. Context recall, on the other hand, measures how well the retrieved contexts align with the ground-truth answer. For context retrieval, the pipeline achieves a context precision and recall of 1, indicating that it successfully retrieves all relevant documents and that the documents retrieved are pertinent to the answer. This, in turn, ensures that the contexts provided to, and the answers given by, the ChatGPT-4o are accurate and useful to those using the system.

**Table 1.** The results for simple questions evaluation using the RAG pipeline and ChatGPT-4o answers

Metric	Average score
Context precision	1.0
Context recall	1.0
Faithfulness	1.0
Answer relevancy	0.935
Harmfulness	0.0
Maliciousness	0.0
Correctness	1.0
Coherence	1.0
Conciseness	1.0

To assess the responses generated by ChatGPT-4o, we also employed several additional metrics to ensure the overall quality of the responses. We first evaluated the responses to assess the faithfulness and relevancy of the answers. Faithfulness measured the factual consistency of the generated answers against the provided context. Our pipeline achieved a score of 1, indicating perfect factual consistency between the generated answers and the provided context, ensuring that all answers generated by ChatGPT-4o were consistent with the information given. Along with this, we assessed answer relevancy, which measured how relevant the answer was to the prompt given by the user. Our pipeline scored 0.935, indicating the answers given were highly relevant to the questions asked by the user. By evaluating this score, we determined that the answers contained all key information, were accurate, and useful.

Moreover, we performed critiques to check different aspects of the answers provided by ChatGPT-4o, including harmfulness, maliciousness, correctness, coherence, and conciseness. For the harmfulness and maliciousness aspects, we evaluated whether the answers contained any content that could cause harm to individuals or groups by promoting harmful actions, providing misleading information, or using language that is intentionally harmful or offensive. We found that the responses generated by our pipeline were neither harmful nor malicious.

We also evaluated the answers for correctness, coherence, and conciseness. The correctness aspect ensured that the answers given were factual and grammatically correct, while coherence ensured that the answers were logically structured and clear, and conciseness ensured the answers were free from redundant or unnecessary information. We found that our pipeline produced responses that were correct, coherent, and concise. By evaluating these different aspects, we ensured that the answers generated by our pipeline did not contain any harmful or malicious content and that all answers were clear and easily understood.

#### 4.3.3. Answer correctness and semantic similarity

While we checked the various aspects and relevancy of the answers in the previous section, in this section, we delved deeper into analyzing the answers produced by ChatGPT-4o to ensure that they were correct and semantically similar to the ground-truths. These results are shown in Table 2.

**Table 2.** The results for the evaluation of the readability of the answers to simple questions using the RAG pipeline and ChatGPT-4o

Metric	Average score
Answer correctness	0.771
Answer semantic similarity	0.970

We began by evaluating the answer correctness, which measured the overall accuracy of the answer when compared to the ground truth, using both semantic similarity and factual similarity. Our pipeline scored 0.771, demonstrating a strong alignment in both factual and semantic similarity between the ground-truth and the generated answer. Furthermore, we also evaluated answer semantic similarity. Answer semantic similarity refers to how well the generated answer aligns with the ground-truth answer. Here, our pipeline scored 0.970, indicating a high level of semantic alignment between the ground truth and the generated answer. This not only demonstrated the overall technical accuracy of our system but also showed attentiveness to details by focusing on specific aspects of the users’ questions. This attentiveness ensured that the answers provided were both correct and closely connected to the users’ queries. By utilizing these metrics, we ensured that the answers provided were both reliable and contextually appropriate.

## 5. Discussion, conclusion, and outlook

The burden of mental disorders, including psychosocial disability, has been escalating globally, underscoring the critical need for effective research and interventions in this area. Our study focuses on leveraging computational text mining, particularly advanced techniques such as word embedding and Large Language Models (LLMs), to extract valuable insights from the vast repository of scientific literature available on PubMed in the fields of psychosocial rehabilitation and psychosocial disability. By harnessing this extensive collection of articles, we aim to enhance our understanding of diagnostics, preventative measures, treatment strategies, and epidemiological factors related to mental health.

Our study proposes a computational text-mining framework to address these challenges by systematically analyzing the vast volume of scientific literature on psychosocial rehabilitation and psychosocial disability. We aim to extract current knowledge, identify trends, and uncover hidden patterns within the literature through dataset access, preprocessing, and computational text mining using advanced techniques. Furthermore, scientific visualization techniques will translate these findings into easily interpretable representations, facilitating knowledge discovery and informing future research and policy interventions.

The proposed computational text mining pipeline presents a promising strategy for extracting facts and knowledge from vast repositories of scientific literature, such as PubMed. However, this study also carries some limitations. For example, the scope of our study is restricted to the available literature within PubMed, potentially excluding relevant sources from other databases. Also, such an automated pipeline may introduce biases in the context of psychosocial disability research. For instance, a study discussing “cognitive-behavioral therapy (CBT)” might end with positive outcomes, but another study referring to the same intervention as “behavioral therapy” might not be recognized as relevant by the algorithm due to differences in terminology. Moreover, the interpretation of extracted insights and trends from computational text mining techniques may require manual validation by domain experts, introducing subjectivity and resource constraints. Despite these limitations, our study aims to provide a valuable foundation for future research in leveraging computational methods to advance understanding and interventions in psychosocial rehabilitation and psychosocial disability research. Accurate identification of the actual scope of psychosocial disability and rehabilitation options relevant for affected individuals might be a limitation of this study. Presently, data on the subject remains a challenge. Our study may offer opportunities to utilize and translate the data for improved social and health outcomes.

Psychosocial disability is a controversial term because people disagree on which mental health conditions count as disabilities. As a result, individuals affected by these conditions often have to prove their disability to access psychosocial rehabilitation. Additionally, societal misconceptions and stigma about mental health conditions remain a public health issue, limiting access to services for those affected (Ebuenyi, 2019; Felix, 2021). The lack of research on this topic has been noted before. Our study uses AI to systematically search and analyze large amounts of data on psychosocial disability and rehabilitation. This approach aims to improve understanding and access to services for affected individuals. Our findings have significant implications for the well-being and clinical outcomes of people with psychosocial disabilities, as well as providing valuable data for policy advocacy and interventions.

Moving forward, our study outlines several promising avenues for future research in the domain of computational text mining for psychosocial rehabilitation and psychosocial disability research. First, broadening the scope of our analysis to include diverse datasets and sources beyond PubMed, for example, PLOS, could yield a more comprehensive understanding of the problem. This expansion could also incorporate additional datasets such as clinical trial repositories, electronic health records (EHRs), and specialized mental health datasets, thus we can enrich the breadth and depth of our insights. Moreover, integrating advanced machine learning approaches for predictive modeling or sentiment analysis could provide valuable foresight into emerging trends in sentiment within the field. Predictive modeling could help identify potential future developments and challenges in psychosocial rehabilitation, while sentiment analysis could reveal how public and professional opinions are evolving over time. Furthermore, fostering interdisciplinary collaborations between researchers, healthcare professionals, and policymakers is crucial. Such collaborations would facilitate the translation of extracted insights into actionable interventions, ultimately improving outcomes for individuals with psychosocial disabilities. By working together, these stakeholders can develop shared decision-making processes and evidence-based policies that are informed by the latest research findings.

## Data Availability

You can access the code and results through our GitHub repository.^2^ This GitHub repository is publicly and freely available for only academic, research, and educational purposes.

## Footnotes

1
https://docs.ragas.io
