# Supplementary material for: Advancing psychosocial disability and psychosocial rehabilitation research through large language models and computational text mining
Source: Glob Ment Health (Camb). 2024 Dec 13;11:e123. doi: 10.1017/gmh.2024.114 (PMC11704382; doi:10.1017/gmh.2024.114)
Supplement: Peer review history

# Editor report: Recommendation: Advancing psychosocial disability and psychosocial rehabilitation research through large language models and computational text mining — R0/PR2

**DOI:** 10.1017/gmh.2024.114.pr2

**Affiliations:** Primary Healthcare Sciences, University of Zimbabwe Faculty of Medicine, Zimbabwe

No accompanying comment.

# Editor report: Decision: Advancing psychosocial disability and psychosocial rehabilitation research through large language models and computational text mining — R0/PR3

**DOI:** 10.1017/gmh.2024.114.pr3

**Affiliations:** Johns Hopkins University Bloomberg School of Public Health, United States

No accompanying comment.

# Editor report: Recommendation: Advancing psychosocial disability and psychosocial rehabilitation research through large language models and computational text mining — R1/PR5

**DOI:** 10.1017/gmh.2024.114.pr5

**Affiliations:** Primary Healthcare Sciences, University of Zimbabwe Faculty of Medicine, Zimbabwe

No accompanying comment.

# Editor report: Decision: Advancing psychosocial disability and psychosocial rehabilitation research through large language models and computational text mining — R1/PR6

**DOI:** 10.1017/gmh.2024.114.pr6

**Affiliations:** Johns Hopkins University Bloomberg School of Public Health, United States

No accompanying comment.

# Editor report: Recommendation: Advancing psychosocial disability and psychosocial rehabilitation research through large language models and computational text mining — R2/PR8

**DOI:** 10.1017/gmh.2024.114.pr8

**Affiliations:** Primary Healthcare Sciences, University of Zimbabwe Faculty of Medicine, Zimbabwe

No accompanying comment.

# Editor report: Decision: Advancing psychosocial disability and psychosocial rehabilitation research through large language models and computational text mining — R2/PR9

**DOI:** 10.1017/gmh.2024.114.pr9

**Affiliations:** Johns Hopkins University Bloomberg School of Public Health, United States

No accompanying comment.
